# Enamel Surface Damage following Debonding of Ceramic Brackets: A Hospital-Based Study

**DOI:** 10.1155/2021/5561040

**Published:** 2021-05-06

**Authors:** Neelutpal Bora, Putul Mahanta, Deepjyoti Kalita, Sangeeta Deka, Ranjumoni Konwar, Chiranjita Phukan

**Affiliations:** ^1^Dentistry, Assam Medical College and Hospital, Dibrugarh 786002, Assam, India; ^2^Forensic Medicine and Toxicology, Assam Medical College and Hospital, Dibrugarh 786002, Assam, India; ^3^Microbiology, All India Institute of Medical Sciences, Rishikesh, Uttarakhand, India; ^4^Radiology, Fakhruddin Ali Ahmed Medical College and Hospital, Barpeta, Assam, India; ^5^Medicine, Tezpur Medical College and Hospital, Tezpur, Assam, India

## Abstract

**Methods:**

The current study includes 80 extracted premolars of human from the patient visiting for orthodontic treatment of Coorg Institute of Dental Sciences, Karnataka, India. The brackets were debonded using four different methods. The enamel surface damage after the procedure was assessed with the Enamel Surface Index (ESI); similarly, the Adhesive Remnant Index (ARI) score was used to determine the adhesive residual deposit. Scanning electron microscopy (SEM) was used to visualize better microporosities and micromechanical retention of adhesive remnants on the enamel surface. The normality of the data was tested using the Kolmogorov–Smirnov test. Depending upon the normality test result, the one-way ANOVA test or Kruskal–Wallis test was used to test the mean ESI and mean ARI differences among different debonding methods along with the appropriate post hoc tests. The necessary ethical clearance was obtained from the Ethics Committee of the institute.

**Results:**

The ultrasonic scaler (US) technique led to more significant enamel surface damage, with 13 (65%) samples in the ESI scores III and IV against the satisfactory surface in 2 (10%) samples with the ligature cutter (LC) technique (ESI-I) reflecting LC as a better technique. The ESI scores (III and IV) for debonding plier (DP) and thermal method (TM) reflected a higher value in 12 (60%) and 10 (50%) samples and caused more damage to the enamel surface as compared to the LC technique. The ARI score was highest (ARI-1 = 40%) with the LC technique, followed by the US (ARI-1 = 20%), TM (ARI-1 = 15%), and DP (ARI-1 = 5%) methods. We have observed a significant association (*p* value <0.05) of the ARI score among four different debonding ways in terms of each tooth's residual adhesive after the bracket removal.

**Conclusion:**

The result establishes the LC technique as a more acceptable one as it causes minimal harm to the debonded surface. The adhesive left on the debonded area is also minimum as compared to the other three methods tested. Therefore, it can be suggested as an ideal method.

## 1. Introduction

A beautiful smile to every patient is the expectation for each orthodontic professional after the debonding procedures. Still, it is likewise essential to have a convenient appearance during the treatment procedures. Therefore, pleasing aesthetics and optimal technical performance should always be there with the ideal orthodontic appliances.

Different researchers have made several efforts to meet up the challenges. Brackets made up of plastic (polycarbonate) were used in the early 1970s as the aesthetic alternative to metal brackets. These brackets lost favour rapidly due to their discolouration and slot alteration triggered by water absorption [1–4]. Those inconveniences have led the scholar to modify the plastic brackets by reinforcing the slots with metal and ceramic fillers [5]. However, the quantifiable hitches such as distortion and discolouration persisted despite those changes.

Ceramic brackets were introduced in the orthodontic speciality in the mid-1980s, which has created a radical change in orthodontic ornamentation [6]. Those ceramic brackets can withstand orthodontic forces and resist staining, unlike plastic brackets. Still, ceramic brackets have low faults as they cannot form chemical bonds with resin adhesives, low breakage hardiness, and increased frictional resistance between metal archwires and ceramic brackets [7–9].

As a further alteration, a newer ceramic bracket with a metal-lined archwire slot was acquainted with the market to lessen some of the difficulties faced in clinical practice. The stainless-steel slot has given the additional advantage to mitigate the increased friction subsequent from the archwires connecting ceramics. This metal slot also reinforces the ceramic bracket to endure routine orthodontic twisting forces [6]. Presently, several ceramic brackets are available for clinical uses. Their popularity and clinical benefits are increasing in contemporary orthodontics with varied usefulness.

Compared to the conventional stainless-steel brackets, ceramic brackets have a superior aesthetic quality, preferred by many patients [10]. However, different reviews reported a breakdown of the ceramic brackets at the time of the debonding process because of their fragile character. With the improper techniques, damages to the enamel surface, pain, and time consumption were also reported [11–14] in many such studies.

Due to the increased patient's necessity, a healthy demand compels for better and comfortable newer devices. Agreeing with the ceramic brackets' distinctive aesthetic nature, various researchers compared the usefulness among different techniques with contradictory findings [4] to find out for the best appliance.

Therefore, we aim to determine the practicality of various debonding techniques to propose a standard practice to minimize damages to the enamel surface with SEM's help.

No significant difference in enamel surface damage among the four debonding methods was one of the null hypotheses (H0) of the present study. Another null hypothesis (H0) was no significant differences in adhesive remnants on the debonded surfaces and bond failure sites among the four debonding methods.

## 2. Materials and Methods

The study was hospital-based and included 80 extracted premolars as samples obtained from those attending the Orthodontic Department of Coorg Institute of Dental Sciences. The sample size was determined using G∗Power 3.1 software with a 5% level of significance, power = 80%, and effect size 0.38. The samples without carious lesion and unrestored premolars were included for this study. The broken samples and sample with morphological anomalies were excluded from this study. 1 : 10 dilution (10%) of 100% formalin (Vishal Chemical of Maharashtra, India) in water was used to store the samples for two weeks. The pumice powder (Neelkanth Ortho Dent Pvt., Ltd., Jodhpur, Rajasthan, India) and a polishing brush (I-Herdsman, Zhengzhou, China) were used for polishing the bonding surface for a minute.

The enamel surface to be bonded was etched with 37% phosphoric acid gel (3M ESPE, Scotchbond Etchant of 3M India Co., Ltd., Bangalore, Karnataka, India) and then washed and dried for 20 seconds. Roth prescription ceramic brackets (Captain Ortho, USA) were bonded to the enamel surface using a primer (Ortho Solo, universal bond enhancer, Ormco, USA) and light cure adhesive, Enlight (Ormco, USA). The light cure unit of Halogen, Dentsply International [15], was used for a blue spectrum of visible light for half a minute. The sample was then stored in 0.9 saline solution for 24 hours to simulate oral condition [12]. The comparison and analysis were made among the four different debonding techniques mentioned as follows:

- Piezoelectric ultrasonic scaler (PUS), straight chisel, Satelec, V. Dent Dental Instrument Co., Ltd., China.
- Debonding plier (DP), Jaypee, India.
- Ligature cutter (LC), Lancer Ortho, Germany.
- Thermal method (TM), hairdryer, Black and Decker, China.

Debonding pliers and ligature cutting pliers applied a squeezing force at the bracket base in a mesiodistal direction for debonding. Satelec piezoelectric ultrasonic scalar with straight tips was used at the bracket adhesive junction for debonding in the ultrasonic method. Bracket removal with the straight chisel tip started at the bracket's incisal margin with the chisel's bevel directed toward the bracket, rather than the enamel surface, to minimize enamel damage. Hair dryer (Philips HP8100/46 Hair Dryer of Philips Electrical Co., Pvt., Ltd., Kolkata, India) was used as a thermal debonding method that acts as a clinical aid debracketing ceramic bracket. The dryer is held 3 mm away from the tooth, and the heated air is directed at the bracket for 15 seconds. After this, the bracket is removed with the aid of a debonding plier. The temperature generated by the dryer is 65^o^C. The total 80 teeth were randomly allocated to four groups for debonding based on the method we used, keeping a 1 : 1 ratio of sample size among all groups.

### 2.1. Enamel Surface Index (ESI)

The current study uses the ESI system to evaluate the damages of areas debonded, introduced by Krell et al. [16]. The four different scores used are as follows:

  Score I: it indicates a satisfactory surface. Fine scratches and some perikymata may also be present.
  Score II: it indicates an acceptable surface. Several marked and some deeper scratches with no perikymata are the features.
  Score III: it means imperfect surface. Several distinct deep and coarse scratches and no perikymata are the features.
  Score IV: it is an unacceptable surface. Coarse scratches and deeply marred appearance are the features.

### 2.2. Adhesive Remnant Index (ARI)

Similarly, the ARI system was used to assess adhesive remnants on the debonded surfaces and bond failure sites, a four-scale index introduced by Bishara et al. [4].

The present study uses scanning electron microscopy (SEM) of Cambridge Instrument Leica (SPI Supplies, USA) company and StereoScan 360 modal to generate electro-micrographs with a magnification of 250 X. It visualizes the microporosities and can detect micromechanical retention of adhesive remnants to suggest debonded areas' damages.

**Figure 1:**
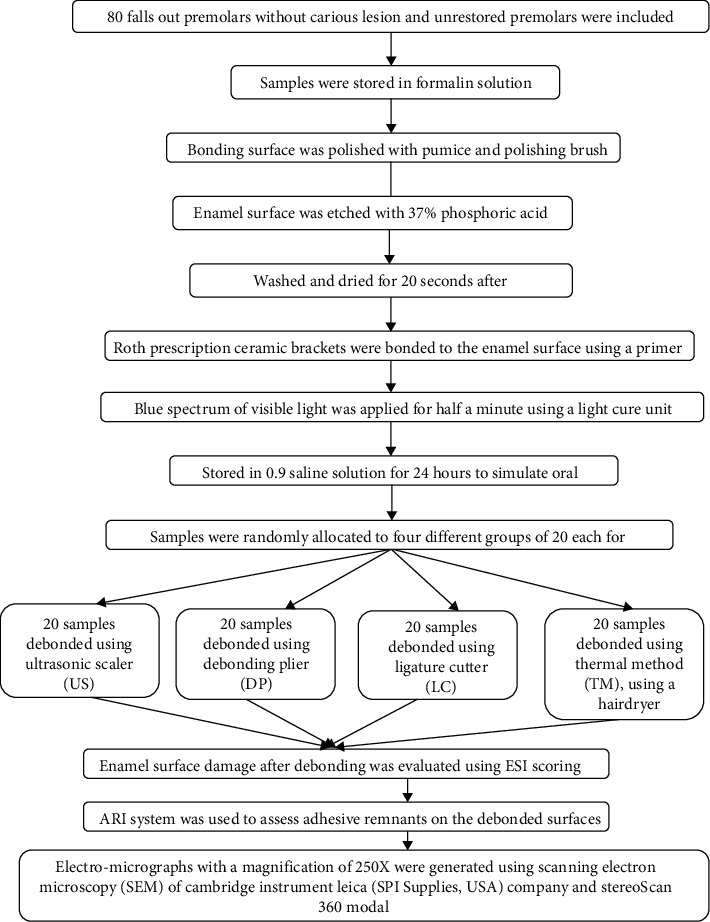
Representative diagram of study outline and procedures.

The outline of the study is presented in Figure 1.

### 2.3. Statistical Analysis

The data were tested for normality using the Kolmogorov–Smirnov test. The association between the debonding techniques with both enamel surface damage and ARI was tested using a chi-square test. The mean ESI and mean ARI differences among different debonding methods were tested using the one-way ANOVA test with Tukey's HSD post hoc test or Kruskal–Wallis H test with Dunn's post hoc test as per the normality of the data. The data were analyzed using Microsoft Excel (Microsoft Corporation, Redmond, WA) and the Statistical Package for the Social Studies (SPSS) version 22 (IBM Corp., Armonk, New York). A *p* value <0.05 was considered to be significant. The prior ethical clearance was obtained from the Ethics Committee of the Coorg Institute of Dental Sciences (Ref No. CIDS/EC/1310).

## 3. Results

The Kolmogorov–Smirnov test results suggested that the data do not follow the normal distribution. Hence, nonparametric tests were performed to analyze the data. As per the ESI score (III and IV) of four different debonding techniques, the US method causes more harm to the debonding surface comprising 13 (65%) cases. In contrast, the enamel surface damage is low with more satisfactory surfaces with the LC method (ESI-I) containing 10%. However, no significant association was observed in enamel surface damage among the four debonding techniques (*p* value = 0.536), as shown in Table 1.

The mean (±SD) value for the 20 brackets debonded by the US method was 2.7500 (0.78640). The Kruskal–Wallis test revealed that the mean ESI score differences among these four debonding techniques were not significant (*p* value = 0.94), as shown in Table 2.

ARI score to determine each tooth's residual adhesive after bracket removal following four different debonding methods reveals a significant association between ARI and the four different debonding techniques (*p* value <0.001). The differences of ARI among other methods were substantial, with maximum cases with LC (ARI-I 40%), as shown in Table 3, where the score was high (ARI-III 65%) with the DP method.

The mean (±SD) ARI values for DP, LC, and TM were 2.6000 (0.59824), 2.0000 (0.91766), and 2.4000 (0.75394), respectively. The mean ARI value for the US method was the least among all. The mean ARI values were significantly different among the four debonding methods (*p* value = 0.002), as shown in Table 4.

Dunn's multiple comparisons of the various debonding techniques revealed that the mean ARI values of the US method are significantly different from that of DP (*p* value <0.001) and TM (*p* value = 0.006). The mean ARI value of the DP method is also considerably different from that of the LC method (*p* value = 0.017), as shown in Table 5.

### 3.1. Use of Scanning Electron Microscopy (SEM)

**Figure 2:**
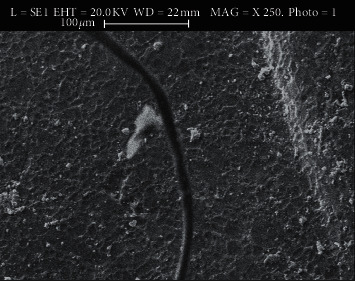
Electro-micrograph showing microporosities and micromechanical retention of adhesive remnants in US method.

**Figure 3:**
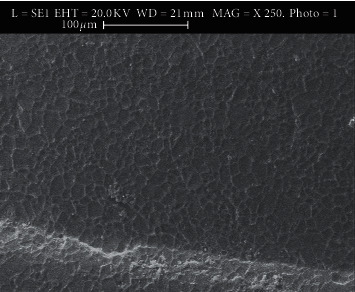
Electro-micrograph showing microporosities and micromechanical retention of adhesive remnants in DP method.

**Figure 4:**
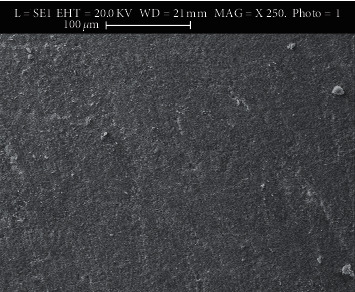
Electro-micrograph showing microporosities with minimal micromechanical retention of adhesive remnants in LC method.

**Figure 5:**
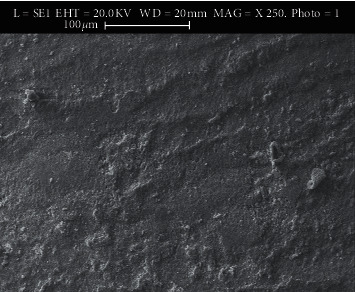
Electro-micrograph showing microporosities with micromechanical retention of adhesive remnants in TM method.

The SEM images show microporosities with micromechanical retention of the adhesive after applying different methods. Figures 2–5 show SEM images showing the enamel damages on its surface with various debonding techniques at 250X magnification.

## 4. Discussion

In the current study, ceramic brackets were considered as presented to the dentistry to meet the increased demand for attractiveness and better appropriateness, as suggested with some reviews [14, 17]. In the line of research [18], the brackets introduced after due treatment were removed mechanically, including the left out adhesive, subsequently, the procedure to eliminate the accumulated dental plaque and discolouration produced during the processes. In considering the adhesive removal, enamel damages were assessed in this present study. The increased enamel roughness was due to scratching resulting from etching, grinding, and consequent polishing which are some variables to be considered while the enamel damages are assessed.

The enamel loss in this study, evaluated by the ESI system, was different among the four methods used. Other surveys reporting enamel damage have provided various enamel loss due to the application of diverse technique [14, 19, 20] agrees the current results. The present study revealed that the US method use led to more significant enamel surface damage among all other methods which agrees with another research outcome [21]. Contradicting the said results, some other researchers [22, 23] mentioned that enamel damage and bracket failure are less with the ultrasonic debonding technique. This fact may be due to the different sample sizes and diverse methodology used. KJS ultrasonic tips and Cavitron ultrasonic scaler in some studies [15, 21, 24] also reported enamel damages. This technique also generates heat on the enamel top that requires water to cool for reducing damaging effects on the pulp tissue. The abovementioned studies support the current result revealing the US method as more inconvenient and unfavourable on the ground of more enamel damages.

In contrast, the enamel surface damage is low with more satisfactory surfaces with the LC method. However, no significant association was observed in enamel surface damage among the four debonding methods (*p* value = 0.536). These insignificant differences in the current study relating to enamel surface damage following different techniques may be due to the smaller sample sizes included in the present study. The mean ESI score differences among those debonding techniques were also not substantial (*p* value = 0.94) which agrees with some reviews [23–25].

The residual adhesive after the bracket removal following the debonding methods in the current study reveals a significant association of the ARI score between all techniques used (*p* value <0.001). The ARI-III score with the DP method was significantly greater than that of the other three techniques which agrees with some reviews [22–24, 26], reflecting all adhesives left on the enamel surface with a distinct imprint of the bracket's mesh.

The LC method's ARI-I score is high, with 8 (40.0%) cases compared to the other three techniques and lowest in DP, revealing better acceptability. It causes minimal enamel damage compared to different approaches during the adhesive removal after the treatment agrees with a research outcome [17].

Many variables could alter the bond efficacy at the enamel-adhesive line, such as the DP method and other prior treatment materials and procedures [27] or the need for particular treatments with a high risk of enamel contamination before bonding used in the current study agrees with a review [28]. Also, these variables should be taken into careful consideration with future studies.

The microporosities with micromechanical retention of adhesive remnants after the bracket removal were assessed by SEM in the current study. Although all the techniques in the present study demonstrated adhesive failure as agreed by a study [27], the LC method shows satisfactory result as revealed in SEM's electro-micrographs. We, the authors of this paper, suggest that in the age of microscopic dentistry, SEM is essential to evaluate enamel damage, which agrees with some surveys [20, 29–34]. SEM in the current study helped better visualizing the microporosities created by orthodontic procedures following bonding that retained adhesive to the enamel structures after the procedures. Thus, it helped to remove the left out adhesive mechanically from the enamel's surface, which agrees with a study [24].

### 4.1. Limitation

Enamel microcracks can be repaired with the recently introduced remineralizing agents, such as casein phosphopeptide-amorphous calcium phosphate [35] and biomimetic hydroxyapatite [36]. Due to the limitation of the objective, this was not followed in the current study. Future research perspective could include these materials in evaluating enamel damage.

Another limitation of the present study was the smaller sample size. A larger sample size would have reduced the possible margin of error in defining a suitable method to minimize damages to the enamel surface, including all permanent teeth, which was not done in the present study.

## 5. Conclusion

The study concludes that the US as a debonding technique causes more enamel surface damage, though no significant differences were observed among different methods. The ARI score also reflects DP as an unfavourable method for debonding. The results reflect the LC method as a better acceptable one that causes minimal enamel damage than other approaches. Further, a significant association between ARI and the four different debonding techniques favoured the LC with higher scores. Hence, LC method as a debonding protocol is suggested for better outcome of the procedures.

## Figures and Tables

**Table 1:** Enamel Surface Index (ESI) scores with the four techniques used for debonding.

ESI score	Debonding techniques					*χ* ^2^ (*p* value)
	US (*n* = 20)	DP (*n* = 20)	LC (*n* = 20)	TM (*n* = 20)	Total (*n* = 80)
I	1 (5.0%)	0 (0.0%)	2 (10.0%)	0 (0.0%)	3 (3.8%)	7.98 (*p* = 0.536)
II	6 (30.0%)	8 (40.0%)	7 (35.0%)	10 (50.0%)	31 (38.8%)	
III	10 (50.0%)	11 (55.0%)	7 (35.0%)	7 (35.0%)	35 (43.8%)	
IV	3 (15.0%)	1 (5.0%)	4 (20.0%)	3 (15.0%)	11 (13.8%)	

Chi-square test.

**Table 2:** Mean Enamel Surface Index (ESI) among different debonding techniques.

Debonding techniques	*n*	Mean	St. deviation	Kruskal–Wallis *χ*^2^	*p* value
US	20	2.7500	0.78640	0.407	*p* = 0.94
DP	20	2.6500	0.58714		
LC	20	2.6500	0.93330		
TM	20	2.6500	0.74516		

Kruskal–Wallis test.

**Table 3:** Adhesive Remnant Index for the methods used.

ARI score	Debonding techniques					*χ* ^2^ (*p* value)
	US (*n* = 20)	DP (*n* = 20)	LC (*n* = 20)	TM (*n* = 20)	Total (*n* = 80)
I	4 (20.0%)	1 (5.0%)	8 (40.0%)	3 (15.0%)	16 (20.0%)	29.75 (*p* < 0.001)
II	16 (80.0%)	6 (30.0%)	4 (20.0%)	6 (30.0%)	32 (40.0%)	
III	0 (0.0%)	13 (65.0%)	8 (40.0%)	11 (55.0%)	32 (40.0%)	

Chi-square test.

**Table 4:** Mean Adhesive Remnant Index among the different debonding techniques.

	*N*	Mean	St. deviation	Kruskal–Wallis *χ*^2^	*p* value
US	20	1.8000	0.41039	15.08	0.002
DP	20	2.6000	0.59824		
LC	20	2.0000	0.91766		
TM	20	2.4000	0.75394		

Kruskal–Wallis test.

**Table 5:** Multiple comparison tests for ARI among different techniques (multiple comparison ARI).

Debonding techniques (I)	Debonding techniques (J)	Mean difference (I−J)	*p* value
US	DP	−0.8000	<0.001
	LC	−0.2000	0.233
	TM	−0.6000	0.006

DP	LC	−0.6000	0.017
	TM	−0.2000	0.415

LC	TM	−0.4000	0.116

Dunn's multiple comparisons tests.

## Data Availability

The data used to support the findings of this study are included in the article.
